# Supplementary material for: IGF-1 Signaling Modulates Oxidative Metabolism and Stress Resistance in ARPE-19 Cells Through PKM2 Function
Source: Int J Mol Sci. 2024 Dec 20;25(24):13640. doi: 10.3390/ijms252413640 (PMC11727907; doi:10.3390/ijms252413640)
Supplement: Supplementary file 1 [file ijms-25-13640-s001.zip › Figure S1.pdf]

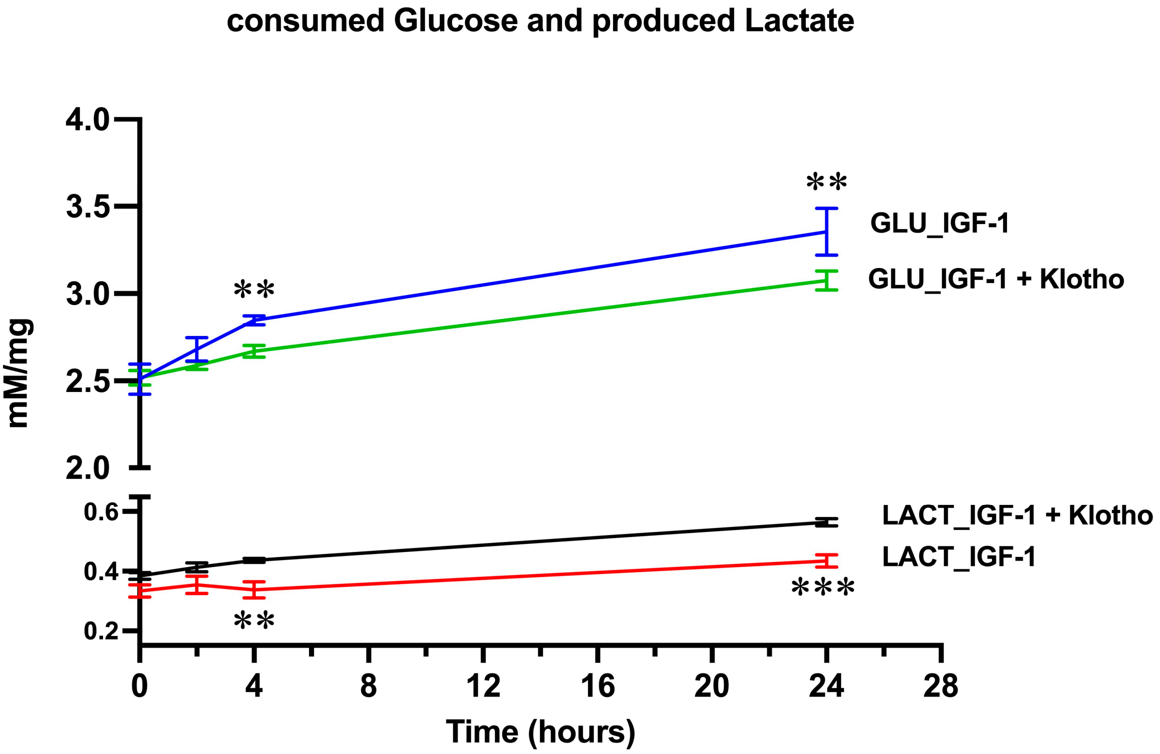

Figure S1. Glucose consumption and Lactate release in ARPE-19 cells treated with IGF-1, in the absence or presence of Klotho.
